# Foreground Detection Based on Superpixel and Semantic Segmentation

**DOI:** 10.1155/2022/4331351

**Published:** 2022-08-31

**Authors:** Junying Feng, Peng Liu, Yong Kwan Kim

**Affiliations:** ^1^School of Intelligent Manufacturing, Weifang University of Science and Technology, Shandong, Weifang 261000, China; ^2^Department of Information and Communication Engineering, Hoseo University, Chungcheongnam-do, Asan 31499, Republic of Korea

## Abstract

Foreground detection is an essential step in computer vision and video processing. Accurate foreground object extraction is crucial for subsequent high-level tasks such as target recognition and tracking. Although many foreground detection algorithms have been proposed, foreground detection in complex scenes is still a challenging problem. This paper presents a foreground detection algorithm based on superpixel and semantic segmentation. It first uses multiscale superpixel segmentation to obtain the initial foreground mask. At the same time, a semantic segmentation network is applied to separate potential foreground objects, and then use the defined rules to combine the results of superpixel and semantic segmentation to get the final foreground object. Finally, the background model is updated with the refined foreground result. Experiments on the CDNet2014 dataset demonstrate the effectiveness of the proposed algorithm, which can accurately segment foreground objects in complex scenes.

## 1. Introduction

**Figure 1:**
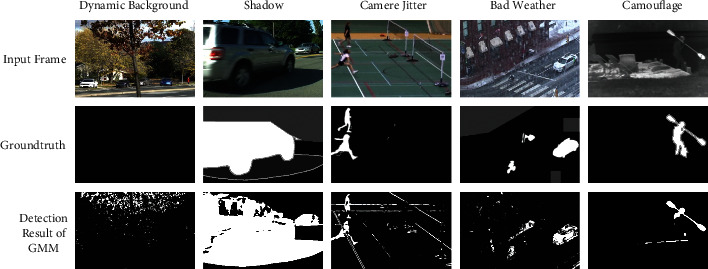
Some challenging scenes for foreground detection.

Foreground detection is a fundamental lower-level task of computer vision. The performance of foreground detection directly affects the performance of a series of subsequent assignments, like object recognition and tracking. The primary purpose of foreground detection is to extract foreground objects from video frames and obtain feature information of foreground objects. Foregrounds are usually moving objects, such as moving human bodies and vehicles. The foreground detection process can be regarded as a classification process of image pixels, looking for differences in continuous image sequences (i.e., videos) and extracting moving objects of interest according to the differences. The moving objects of interest are in the foreground, and the rest are classified as background. The foreground object detection is generally realized by background subtraction, which usually contains unsupervised and supervised algorithms. In recent surveys [1–4], we can find various background subtraction algorithms studied from multiple aspects. Despite the continuous development of computer vision-related technologies, foreground detection has achieved impressive results in some application scenarios. However, foreground detection still faces significant challenges in real-world applications due to various complex interferences in natural scenes, such as dynamic background, shadow, lousy weather, camouflage. From Figure 1, we note that traditional methods often make false positives and negatives.

**Figure 2:**
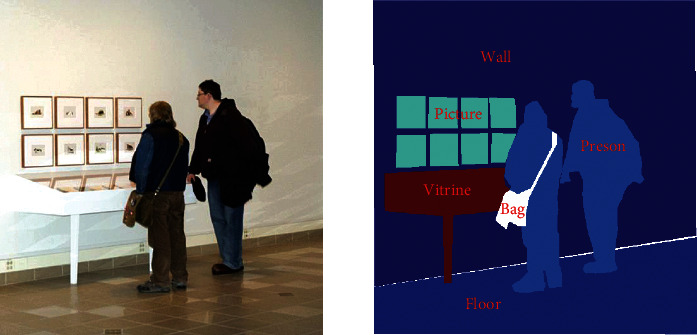
An example of semantic segmentation.

Superpixel is an image segmentation technology proposed and developed by Ren and Malik [5], which refer to irregular pixel blocks with specific visual significance composed of adjacent pixels with similar texture, color, brightness, and other features. The pixels in a superpixel have a more robust connection with each other than pixels in a region. Some applications [6, 7] based on superpixels verify its effectiveness in foreground detection. Semantic segmentation is a deep learning algorithm that associates a label or class with each pixel of an image. It identifies the collection of pixels that make up a different class. It is exemplified in Figure 2. Semantic segmentation shows powerful performance in foreground detection [8]. Based on these motivations, a new approach named superpixel and semantic segmentation foreground detection (SSSFD) is proposed to address the challenges mentioned above. SSSFD can accurately detect moving objects according to the experiments on the CDNet2014 dataset [9]. The contributions of this paper are the following: (1) A background subtraction algorithm for multiscale superpixel segmentation with postoptimization is proposed. (2) A semantic segmentation network is trained on the CDNet2014 subset. (3) The final results combining superpixel and semantic segmentation participate in the background model update in real-time.

The rest of the paper is organized as follows: Section 2 introduces the related works of our proposed method. In Section3, we report the implementations of SSSFD in detail. The experiments and experimental results' qualitative and quantitative analysis are described in Section 4, and finally, this paper is briefly summarized in Section 5.

## 2. Related Works

Over the past 30 years, plenty of foreground detection approaches have been studied. They can be grouped into unsupervised and supervised methods.

### 2.1. Unsupervised Methods

Unsupervised background subtraction algorithm usually combines handcraft features with advanced background modeling rules to achieve foreground separation. The unsupervised background subtraction algorithm usually consists of three parts: the initialization of the background model, the extraction of the foreground region, and the update of the background model.

The first unsupervised algorithm widely used for foreground detection was the Gaussian Mixture Model (GMM) proposed by Stauffer and Grimson [10], which is a statistical-based parametric model. The basic principle of GMM is to represent the distribution of image pixels in the time domain with multiple (3–5) Gaussian models. The central assumption of the GMM is that the pixel obeys a Gaussian distribution. The variance of a pixel determines the pixel class. If the variance of the pixel is higher than a specific value, the pixel is considered a foreground pixel. This method assumes that the variance of the background pixels is in a relatively low position, which is not always accurate in practice. In addition, when the background changes rapidly, it is hard for this method to keep up with the background variations, and the detection of fast-moving objects will result in missed detection and ghost. Moreover, efficiently updating model parameters to match changes in the background is also a challenging problem.

To overcome the problems arising from the above GMM, nonparametric statistical background models were proposed to improve foreground detection performance effectively. The nonparametric estimation background subtraction methods do not need to preassume the model and estimate the model's parameters. The classic methods are Kernel Density Estimation (KDE) [11] and Visual Background Extractor (ViBe) [12]. KDE reads the historical data in the recent samples of each pixel. It calculates the actual probability density of a pixel from the kernel density estimation of the pixel for foreground detection. This method requires a large amount of calculation, the real-time performance is not good enough, and the detection accuracy is average. In some cases, a smear phenomenon will occur. The ViBe is a pixel-level foreground detection algorithm. The key idea of this method is to store a sample set for each pixel in the image. The sample-set consists of the past pixel value of the pixel position and the pixel value of its neighborhood. Determine whether the pixel is a foreground or background point by comparing the sample set of the current pixel. If the pixel meets the characteristics of the sample set, the pixel is the background point. Otherwise, it is the foreground point. The ViBe model uses a random strategy to establish and maintain the background model. ViBe makes use of a time sampling update strategy to reduce the update frequency. This method requires less computation, less memory usage, and faster processing speed but has the disadvantage of ghost. In order to eliminate ghosts and further improve the performance of the ViBe, various algorithms based on ViBe have been proposed. St-Charles et al. [13] presented a pixel-level segmentation method with spatiotemporal binary features and color information. In addition, an adaptive feedback mechanism was used to dynamically adjust parameters such as model fidelity, segmentation noise level, distance threshold, and local update rate. This approach achieved effective and robust results in the most challenging scenes. Lu and Lu [14] proposed a weight-sample-based method using samples with variable weights, minimum-weight update policy, reward-and-penalty weighting strategy, and spatial-diffusion policy for foreground detection. Nonparametric models do not need to specify the distribution model of pixels but still need to use a threshold-based method for each pixel. Due to the different characteristics of various scenes, it is difficult to infer the threshold of each pixel through a unified calculation model.

### 2.2. Supervised Methods

Supervised background subtraction algorithms typically rely on a trained deep neural network (DNN) to segment the foreground from the background. All of these methods are divided into four steps: (1) use a particular algorithm to get a clean background image; (2) generate a dataset of specific scenes for training; (3) exploit the dataset to train the deep neural network; (4) utilize the trained neural network and the background image to perform foreground detection on the image sequence. Supervised methods can achieve good results for foreground detection tasks in many complex scenes, significantly improving the accuracy of the foreground detection.

Braham and Van Droogenbroeck [15] proposed the first background subtraction model ConvNet based on convolutional neural networks (CNNs). Temporal median filtering generates a static background image from 150 consecutive frames. A pair of 27^*∗*^27 image patches is extracted from the background image and the corresponding original image and are input into the constructed CNN model. The output is the foreground probability of the center pixel in this input image patch. Because ConvNet is one of the most straightforward approaches to model the differences between the background and the foreground using CNNs, there are several limitations listed below: (1) the high-level information is challenging to learn through patches [16]; (2) the trained network only works in specific scenes due to the highly redundant data of video frame sequences in training. If the ConvNet is used for other video scenarios, it should be retrained; (3) since ConvNet adopts a pixelwise scheme, treating each pixel as independent, isolated false negatives and false positives may appear in the foreground mask; (4) ConvNet is a deep encoder-decoder network with a generator network. However, one of the drawbacks of this generator network is that the object edges cannot be preserved, leading to the generation of blurry foreground areas. Since the first valuable research, various subsequent algorithms have been proposed to alleviate these limitations. Wang et al. [17] designed a new background model based on CNN with two critical ideas. One is the multiscale structure. For the model to segment foreground objects of different sizes accurately, the original image is down-sampled with 0.75 and 0.5, respectively. Then the sampled image and the original image are used as the model's input. The second is cascaded architecture. In order to reduce pixel misclassification caused by local information, the foreground probability map output by the first multiscale structure and the original image is re-input to the second multiscale structure. The second CNN model further calculates the accurate foreground probability map.

A fully convolutional network (FCN) was first proposed to process the semantic segmentation of an image by Long et al. [18]. The FCN consists entirely of convolutional layers, which convert the fully connected layers of CNNs into deconvolutional layers, thereby preserving the location and semantic information of pixels. FCN can efficiently learn to make dense predictions for per-pixel tasks. Therefore, it is widely used in pixel-by-pixel computer vision tasks such as pixel classification. Based on the traditional FCN with atrous convolution, pyramid scene parsing network (PSPNet) which was added to the PSP module between the encoder and the decoder was presented by Zhao et al. [19]. The PSP module can obtain more global context information, producing high-quality results in scene parsing assignments. DeepLab v1 [20] combines deep convolutional neural networks with conditional random field (CRF), which overcomes the local properties of deep networks and can better locate segmentation boundaries. DeepLab v2 [21] makes full use of atrous convolution, which can effectively expand the receptive field and incorporate more context without increasing parameters. In addition, deep convolution neural networks (DCNNs) are combined with CRF to optimize the network performance further. Moreover, the atrous spatial pyramid pooling (ASPP) module enhances the robustness of the network for multiclass segmentation at multiple scales. It uses different sampling scales and receptive fields to extract input features and capture target and context information at multiple scales. DeepLab v3 [22] designs cascaded or parallel atrous convolution modules and improves ASPP. DeepLab v3+ [23] optimizes segmentation results by adding a simple yet efficient decoder module on top of DeepLab v3. Badrinarayanan et al. [24] proposed a deep network SegNet for semantic segmentation based on FCN and modified VGG-16 [25]. It still adopts the encoder–decoder structure, and the encoder part uses the first 13 layers of a convolutional network of VGG-16. Each encoder layer corresponds to one decoder layer. The output of the final decoder is fed into a softmax classifier, which generates class probabilities for each pixel independently.

In addition to the algorithms mentioned above, other supervised algorithms such as generative adversarial networks (GANs) [26–28], deep CNNs [29–32], structured CNNs [33], and 3D CNNs [34–36] have also achieved good results in foreground detection applications.

## 3. Proposed Approach: SSSFD

**Figure 3:**
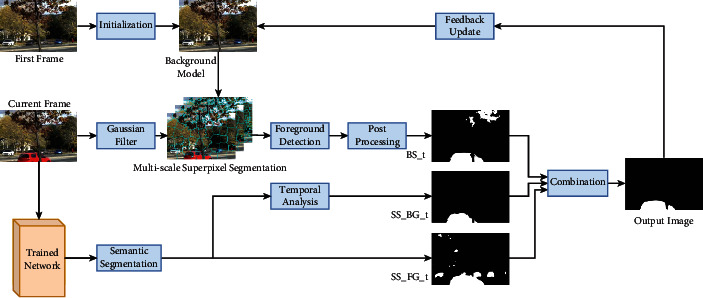
The pipeline of the proposed method SSSFD.

SSSFD combines an unsupervised algorithm based on multi-scale superpixel segmentation (MSS) and a supervised algorithm based on semantic segmentation. The detailed implementation steps are introduced in this section.  Figure 3 shows the pipeline of SSSFD. First, train the semantic segmentation network and then, the background subtraction MSS is performed on the input image to obtain the coarse foreground mask. The process mainly consists of multiscale superpixel segmentation, foreground detection, and postprocessing. At the same time, the semantic foreground segmentation map is obtained by semantically segmenting the input image using the pretrained network. Subsequently, the background subtraction and semantic segmentation results are combined with specific rules to get refined foreground result. Finally, the background model is updated with the elegant foreground result.

### 3.1. Unsupervised Method MSS

#### 3.1.1. Preprocessing

We first apply a Gaussian filter to the current input image. Gaussian filtering is a linear smoothing filter that can eliminate Gaussian noise and is widely used in the noise reduction process of image processing. Gaussian filtering is a process of weighted averaging of the entire image. Each Gaussian filtered image pixel value is obtained by the weighted average of the original image pixel and its neighboring pixels. The template defines the weight and neighborhood.

Next, we use different scales to perform superpixel segmentation on the Gaussian filter image to get foreground probability maps at each scale. The number of pixels in a superpixel block, that is, how many superpixels an image is divided into, is significant. Suppose the size of the superpixel block is large. In that case, the pixels in the superpixel block may contain both foreground and background object pixels, which will reduce the accuracy and sensitivity of the final detection result. If the size of the superpixel block is small, compared with the pixelwise foreground detection algorithms, the accuracy of the last detection result is not significantly improved, and the robustness is poor. Therefore, we perform multiscale superpixel segmentation on video sequences and then execute foreground detection. We use the SLIC algorithm [37] to segment the current frame, and then the segmented superpixel blocks at multiple scales can be expressed as follows:

(1) $$\begin{array}{c} \left\{SP_{1},SP_{2},\ldots ,SP_{K}\right\}=\left\{SP_{i,j}|i\in \left[1,K\right],j\in \left[1,N_{i}\right]\right\}, \end{array}$$

where *i* is the scale subscript used in multiscale segmentation, *K* is the number of multiscales used, *N*_*i*_ is the number of superpixels under scale *i*, and *j* is the corresponding index of the superpixel block under a particular scale.

#### 3.1.2. Foreground Detection

In foreground detection, the feature that distinguishes foreground and background is the RGB color feature. We use improved frame difference (IFD) to compute the difference between two corresponding superpixel patches in the current and background images. The IFD approach is not to calculate the feature values pixel by pixel but to perform a subtraction operation with a region of *R* around a pixel and find the minimum value of the result as the variation of the pixel. This measure can effectively improve the robustness of the background subtraction algorithm and cope with the foreground detection of complex scenes such as dynamic backgrounds. IFD can be defined using the following formula:

(2) $$\begin{array}{c} D\left(x,y\right)=\text{min}\left\|I_{t}\left(m,n\right)-I_{b}\left(x,y\right)\right\|,\;\left(x,y\right)\in SP_{\left(i,j\right)},\;m,n\in x,y\pm R, \end{array}$$

where (*x*, *y*) is the coordinate of a pixel point in the superpixel (*i*, *j*). *I*_*t*_ represents the pixel value of the current frame, *I*_*b*_ represents the pixel value of the background image, (*m*, *n*) is the coordinate of pixels in the neighbor region of (*x*, *y*), and *R* is the radius of the neighbor region.

We use a superpixel instead of a single-pixel for background subtraction. The superpixel, including the similarity and spatial relationship between pixels, can improve the performance of the proposed unsupervised algorithm. The foreground image consists of superpixels with foreground or background labels. Whether each superpixel is marked as foreground or background is determined by the variation in the RGB color characteristics of the superpixel itself. The color feature variation of a superpixel can be calculated by averaging the color feature changes of each pixel that composes the superpixel. This average, in turn, acts as a feature variation value for each pixel in the superpixel. Formula (3) can represent the process:

(3) $$\begin{array}{c} P\left(SP_{i,j_{\left(x,y\right)}}\right)=M_{SP_{\left(i,j\right)}}=\frac{\sum_{x,y\in SP_{i,j}}D\left(x,y\right)}{\left|SP_{i,j}\right|}, \end{array}$$

where *D*(*x*, *y*) is obtained from formula (2), and represents the minimum difference of the feature values between the current frame and the background model in coordinate (*x*, *y*). |*SP*_*i*,*j*_| represents the number of pixels in the superpixel block.

After calculating the average value of superpixel, *M*_*SP*_(*i*, *j*)__ is compared with a threshold *T*_*sp*_ to determine whether the superpixel is foreground or background according to formula (4), so as to obtain the foreground segmentation map at a certain scale.

(4) $$\begin{array}{c} Fg_{i}\left(x,y\right)=\left\{\begin{array}{cc} 1, & P\left(SP_{i,j_{\left(x,y\right)}}\right)>T_{sp}.\\ 0, & \text{otherwise,} \end{array}\right. \end{array}$$

where *Fg*_*i*_(*x*, *y*) means the foreground of the pixel with coordinate (*x*, *y*)  at scale *i*, and *T*_*sp*_ is the threshold to judge whether a superpixel belongs to the foreground or not.

The number of superpixels in an image will affect the results of foreground detection. We use a multiscale strategy to effectively reduce the performance degradation of the algorithm caused by the uncertainty of the number of superpixels and better detect foreground objects. The formula is as follows:

(5) $$\begin{array}{c} Fg\left(x,y\right)=\frac{\sum_{i=0}^{k}Fg_{i}\left(x,y\right)}{K}, \end{array}$$

where *Fg*(*x*, *y*) is the foreground probability of the image, and K is the total number of used scales.

Finally, we compare the foreground probability map with a threshold to obtain the foreground detection result of the image. The formula is as follows:

(6) $$\begin{array}{c} \text{Result}=\left\{\begin{array}{cc} 255, & Fg\left(x,y\right)>T_{fg},\\ 0, & \text{otherwise,} \end{array}\right. \end{array}$$

where *T*_*fg*_ is the threshold for judging the foreground.

#### 3.1.3. Postprocessing

**Figure 4:**
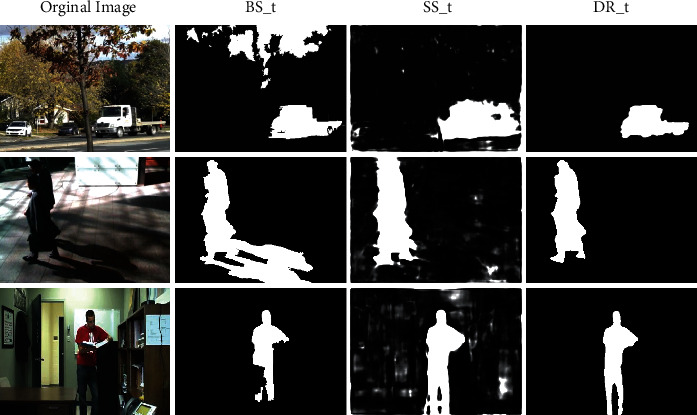
Illustration of MSS results *BS*_*t*_, semantic foreground segmentation results *SS*_*t*_, and SSSFD results *DR*_*t*_.

We use a region-based strategy when using the IFD to calculate the pixel difference between the current frame and the background model. This strategy will lead to the detection result being always slightly larger than the ground truth, called Region of Moving Object Slightly Larger (RMO-SL), as illustrated in [38]. Therefore, we use contour-optimizer (CO) [38] to optimize the background subtraction results to gain the final foreground segmentation map of the unsupervised method. The CO postprocessing method consists of suppression region, edge, and wrong edge detection. Suppression region detection aims to determine areas where foreground moving objects must not appear. Edge detection can generate the edges of the result. The wrong edges are detected by combing suppression regions and caught edges. After eliminating the wrong edges, we get the last outcomes of the MSS *BS*_*t*_, which are shown in the second column of Figure 4.

### 3.2. Train the Network for Semantic Segmentation

We adopt an advanced semantic segmentation network called DeepLabv3plus [23] and pretrain it on the ADE20K dataset [39] to acquire initial weight parameters. ADE20K can be used for scene perception, parsing, segmentation, multiobject recognition, and semantic understanding, with 20210/2000 densely annotated images for training and validation. There are 150 common objects in the dataset, denoted as *C*={*c*_1_, *c*_2_, *c*_3_,…, *c*_150_}. Following the same procedure as [40], we divide these objects into the foreground and background classes. The foreground class is *F*_*c*_={person, car, cushion, box,book,boat, bus,truck,bottle, van,bag, bicycle}, and the background class is *Bc*, *Bc*∩ *Fc*=∅,  *Bc* ⊂ *C*.

Since there is no unified training dataset for foreground detection, we generally choose a certain number (*N*) of frames from CDNet2014 to form a training set when training a semantic segmentation network. There are usually three strategies for selecting frames [17]. One is to select one frame per *M*/*N*, where *M* is the total number of video frames. The second is to choose *N* frames randomly from the video. The third is to choose N frames manually. Considering that the video contains many redundant frames, it will cause the problems of insufficient foreground information and an unbalanced number of positive and negative samples, which will affect the training effect of the network. Therefore, we use the manual selection of frames and select frames that contain foreground objects as much as possible for training, which will improve the accuracy of the network for foreground segmentation. Considering that there are a considerable amount of redundant frames in the video, actively screening valid frames instead of randomly selecting the training data can not only effectively reduce the amount of redundancy but also reduce the severe imbalance problem of positive and negative samples. We employ the dataset Sub-CDNet2014 provided by [41], which consists of 200 frames manually selected from each category of CDNet2014. The Sub-CDNet2014 is a total of 10,600 images, of which 80% are used for training, and the remaining 20% are used for validation. Transfer the pretrained network to Sub-CDNet2014.

Before training, to further improve the performance of the network, we perform data augmentation and positive and negative sample weight calculations on the images in Sub-CDNet2014. Data augmentation adopts standard geometric transformation methods such as inversion, translation. The weights of positive and negative samples are calculated as follows: expand every input ground truth into a corresponding one-dimensional vector, denoted as *P*_1_, *P*_2_,…,  *P*_*N*_, *N* is the number of input ground truth. Calculate the total number of pixels in *P*:

(7) $$\begin{array}{c} {\text{Total}}_{P}=\sum_{t=1}^{N}\sum_{i=1}^{L}P_{t}\left(i\right), \end{array}$$

where *L* represents the number of pixels in an image, and *t* is the time sequence. Compute the classes of distinct pixel values in *P*:

(8) $$\begin{array}{c} C_{t}=\text{unique}\left(P_{t}\right), \end{array}$$

where “unique” means to find the types of different classes in the ground truth. Calculate the frequency of occurrence of each value in each class:

(9) $$\begin{array}{c} H_{t}\left(idx\right)=\text{bincount}\left(P_{t}\right), \end{array}$$

where “idx” represents the class, and “bincount” is used to count the frequency of pixels in each class. Compute the weight value of each class according to the following formula:

(10) $$\begin{array}{c} \text{weight}\left(i\text{dx}\right)=\frac{{\text{Total}}_{P}}{\left(C_{t}{}^{*}H_{t}\left(i\text{dx}\right)\right)}. \end{array}$$

After the training is completed, the trained network is executed on the CDNet2014 dataset to obtain each image's semantic foreground probability map. The probability is mapped to *SS*_*t*_  in the range 0–255 according to formula (11):

(11) $$\begin{array}{c} SS_{t}\left(x\right)=255{}^{*}{\text{map}}_{t}\left(x\right), \end{array}$$

where *x* represents a fixed location of a pixel, and map_*t*_ is the image's semantic foreground probability map.

The third column of Figure 4 shows some semantic foreground segmentation results.

### 3.3. Collaboration Process between Superpixel and Semantic Segmentation

#### 3.3.1. Temporal Analysis of Semantic Background Model

In the semantic segmentation result, *SS*_*t*_, stationary semantic foreground such as cars in a parking lot will be incorrectly classified as the foreground. This problem can be addressed by a similar approach to [8], that is, establishing a semantic background model (SBM). *SS*_*t*_^*FG*^ and *SS*_*t*_^*BG*^ two information with high confidence in foreground and background, respectively, are derived from *SS*_*t*_. *SS*_*t*_^*FG*^ can be used to help reliably detect foreground pixels. At the same time, *SS*_*t*_^*BG*^ provides necessary information for detecting background pixels. They are defined as follows:

(12) $$\begin{array}{c} SS_{t}^{FG}\left(x\right)=SS_{t}\left(x\right)-SBM_{t}\left(x\right), \end{array}$$

(13) $$\begin{array}{c} SS_{t}^{BG}\left(x\right)=SS_{t}\left(x\right), \end{array}$$

where *SBM*_*t*_(*x*) represents the semantic background model at the pixel *x* at time *t*. At first, initialize *SBM*_*t*_(*x*) with the first frame. We then continuously update the semantic background model to adapt to scene variations using a conservative update strategy that alleviates the problem of slow and intermittently moving objects blending into the background model. Specifically, the foreground pixels FG of the final detection result DR are not updated, and the background pixels BG are updated according to the learning rate *∂*.

(14) $$\begin{array}{c} \left\{\begin{array}{c} SBM_{0}\left(x\right)=SS_{0}\left(x\right),\\ SBM_{t+1}\left(x\right)=SBM_{t}\left(x\right),\text{if }DR_{t}\left(x\right)=FG,\\ SBM_{t+1}\left(x\right)=\left(1-\partial \right)*SBM_{t}\left(x\right)+\partial *SS_{t}\left(x\right),\text{ if}DR_{t}\left(x\right)=BG. \end{array}\right. \end{array}$$

**Figure 5:**
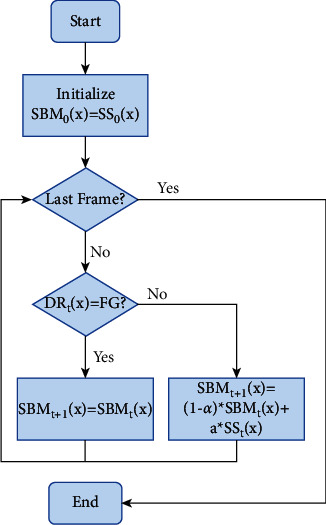
SBM's updating process.

Figure 5 shows the updating process of *SBM*_*t*_(*x*).

#### 3.3.2. Combination Rules

Using the detection results of MSS and semantic segmentation, we can determine the class of a pixel according to the defined rules.

First, in *SS*_*t*_^*BG*^, pixels with a low semantic probability should be classified as background points, regardless of the background subtraction result *BS*_*t*_. Depending on this rule, challenges such as dynamic backgrounds, illumination variations, and cast shadows that seriously affect the performance of unsupervised foreground detection algorithms can be solved, and false positives in such scenarios can be greatly reduced, as shown in the first and second rows of Figure 4.

(15) $$\begin{array}{c} DR_{t}\left(x\right)=0,\;\text{if}SS_{t}^{BG}\left(x\right)\leq Th_{BG}, \end{array}$$

where *Th*_*BG*_ is the decision threshold value of background pixels.

Second, in *SS*_*t*_^*FG*^, pixels with high-semantic probability should be classified as foreground points. This rule reduces holes in foreground objects due to occlusion or model absorption, which tends to be false negatives, as shown in the third row of Figure 4:

(16) $$\begin{array}{c} DR_{t}\left(x\right)=255,\;\text{if}SS_{t}^{FG}\left(x\right)\geq Th_{FG}, \end{array}$$

where *Th*_*FG*_ denotes the decision threshold value of foreground. Specify *Th*_*FG*_ > *Th*_*BG*_.

Finally, if the value of a pixel does not meet the above two conditions, the semantic segmentation result cannot provide enough decisions to determine the classification of the pixel. At this time, we directly use the MSS result *BS*_*t*_ as the pixel class:

(17) $$\begin{array}{c} DR_{t}\left(x\right)=BS_{t}\left(x\right). \end{array}$$

The complete collaboration process is summarized in Table 1.

In brief, this process can also be represented by the following formula:

(18) $$\begin{array}{c} DR_{t}\left(x\right)=\left\{\begin{array}{cc} BG, & \text{if }SS_{t}^{BG}\left(x\right)\leq Th_{BG},\\ FG, & \text{elseif }SS_{t}^{FG}\left(x\right)\geq Th_{FG},\\ B_{t}\left(x\right), & \text{otherwise}. \end{array}\right. \end{array}$$

### 3.4. Background Update

The background update is significant. A timely and accurate update can make the algorithm better cope with complex scene changes and improve the robustness and accuracy of the algorithm. We adopt different update strategies for the pixels detected as foreground or background.

**Figure 6:**
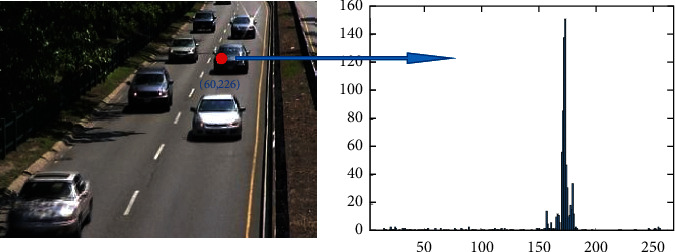
The background update of the foreground pixels in foreground detection.

If a pixel is detected as a background pixel during foreground detection, the pixel value of the corresponding pixel in the background model is directly replaced with the current pixel value. We use a statistical histogram model to update the foreground pixel if a pixel is detected as foreground.  Figure 6 shows the histogram statistics of pixel values for a pixel within a certain period. The abscissa is the pixel's gray value at different times, and the ordinate is the frequency of occurrence of the gray value in this period. We utilize the statistical histogram's peak to replace the background model's pixel at the same location as the foreground pixel of the current frame The process is denoted as follows:

(19) $$\begin{array}{c} p_{bm}=\tilde{M}\left(P\right),P=\left\{p_{1},p_{2},\ldots ,p_{t}\right\}, \end{array}$$

where *p*_*bm*_ is the pixel value in the background model at the same position as the foreground pixel of the current frame, and $\tilde{M}$ represents the mode operation that computes the max value in a set, and P is the set of pixel values in the time sequence from 1 to *t*.

The detailed procedure of the SSSFD algorithm for detecting moving objects is summarized in Algorithm 1.

## 4. Experiments

### 4.1. Dataset and Evaluation Metrics

CDNet2014 [9] dataset is currently the most extensive video dataset with pixel-level labels. It consists of 53 real-world scenarios in 11 different categories. All the video sequences are captured by fixed cameras. This paper runs our proposed algorithm on five categories of the CDNet2014 dataset, evaluates the algorithms' performance, and compares them with other representative foreground detection methods.

During the quantitative evaluation, we choose recall, precision, and *F*-measure [42] to evaluate the performance of the proposed approach. The recall is the detection rate, indicating the percentage of pixels correctly classified as foreground than foreground pixels in the ground truth. Precision is the accuracy rate, meaning the proportion of detected true positives to the number of all detected positives. *F*-measure is the weighted harmonic average of recall and precision, which is the comprehensive evaluation metric that can best reflect the performance of the foreground detection algorithm. The higher values of recall, precision, and *F*-measure, the better performance of the algorithm is. They are defined as follows:

(20) $$\begin{array}{c} \text{Recall}=\frac{\text{TP}}{\text{TP}+\text{FN}},\\ \text{Precision}=\frac{\text{TP}}{\text{TP}+\text{FP}},\\ F-\text{measure}=2\times \frac{\text{Recall}\times \text{Precision}}{\text{Recall}+\text{Precision}}, \end{array}$$

where TP, FP, TN, and FN are true positive, false positive, true negative, and false negatives, respectively.

### 4.2. Experimental Results and Analysis

This section executes several experiments on the CDNet2014 dataset to analyze the proposed SSSFD algorithm in detail. We conducted an ablation study of SSSFD and performed qualitative and quantitative comparisons with other fundamental and state-of-the-art foreground detection algorithms to evaluate SSSFD. In this work, all experiments are implemented on a PC with a 3.6 GHz CPU, 16G RAM, and NVIDIA GeForce RTX 2080 Ti GPU in the Matlab 2020a environment under Windows 10.

A few hyperparameters should be set before initializing a background model, as shown in Table 2.

#### 4.2.1. Ablation Study for SSSFD

**Figure 7:**
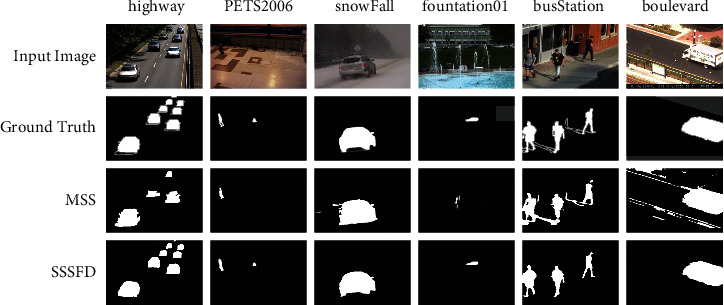
Visual results for MSS and SSSFD.

**Figure 8:**
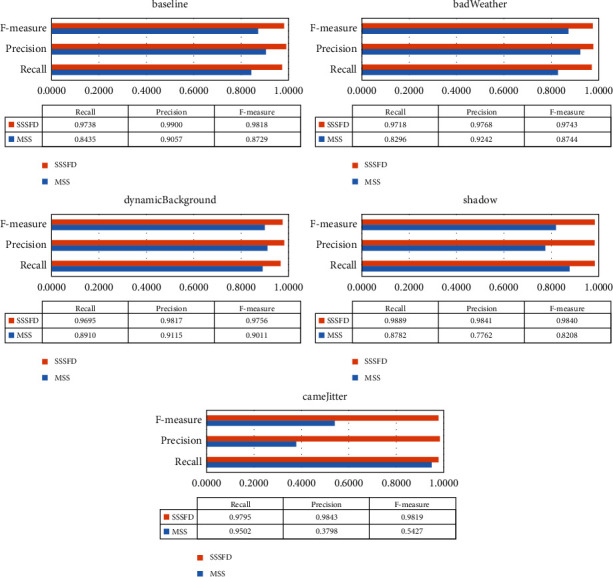
Performance comparison results of SSSFD and MSS in baseline, bad weather, dynamic background, shadow, and camera jitter video sequences.

Figure 7 presents the visual image comparison of MSS and SSSFD, and Figure 8 reports the quantitative comparison results of SSSFD and MSS in some scene categories of the CDNet2014 dataset. From the chart, we can note that the SSSFD algorithm that combines superpixel and semantic segmentation outperforms the superpixel-only foreground detection algorithm MSS in all categories.

SSSFD improves the *F*-measure by a significant boundary of 12.5% on the baseline category. This is mainly because the semantic segmentation network can completely segment potential foreground objects encountered in the training process. It is given a lift to *F*-measure by improving the foreground detection rate and more correctly recalled foreground pixels. SSSFD provides around 11.4% improvement compared to MSS in the bad weather category. MSS may be wrongly detected large snowflakes as foreground. Semantic segmentation can eliminate this false detection, reduce false positives, and improve precision and *F*- measure. Since MSS exploits the RGB color feature, it is easy to detect moving shadows as foreground. The high-performance semantic segmentation network can more accurately determine the shadow position through the extracted global information, eliminating the shadow. SSSFD also has a remarkable improvement relative to MSS in the shadow category, around 19.9%.

Dynamic background category is the different kind of challenge as camera jitter. There is the movement of some background objects in the dynamic background, such as curtains moving with the wind, swaying leaves, and sparkling water. The camera jitter video background contains no moving objects. The position of the entire camera changes irregularly due to external forces (such as wind blowing, vibration), resulting in the blurred video. Camera jitter will cause the pixels on the object's edge to be detected as foreground points in the previous frame, the current frame as background points, and the next frame may be detected as foreground points. In the “fountain01” video sequence with dynamic background, the MSS algorithm detects the gushing fountain as the foreground while missing small natural moving objects, making this sequence detection fail. When the SSSFD is adopted, the false foregrounds are eliminated, and the correct ones are recalled. The F-measure has a surprising improvement on this sequence. The improvement in *F*-measure across the dynamic background category is 8.3%. Although MSS can recall most of the foreground moving object pixels in the camera jitter category, some false foreground pixels are also identified simultaneously due to drastic camera jitter, resulting in low precision of recalled pixels. However, SSSFD will segment the correct foreground object for each input frame, ignoring camera shake. Compared with MSS, the F-measure value of SSSFD is improved by an astonishing 80.9%.

The MSS is removed from SSSFD, and the quantitative foreground results obtained by the semantic segmentation network (SSN) and SSSFD are compared, as shown in Table 3. Each metric of SSSFD is better than SSN, which proves the role of MSS in SSSFD. The pixels that the semantic segmentation is not sure whether it is background or foreground, can be determined the class by MSS.

#### 4.2.2. Comparison with Other Algorithms

**Figure 9:**
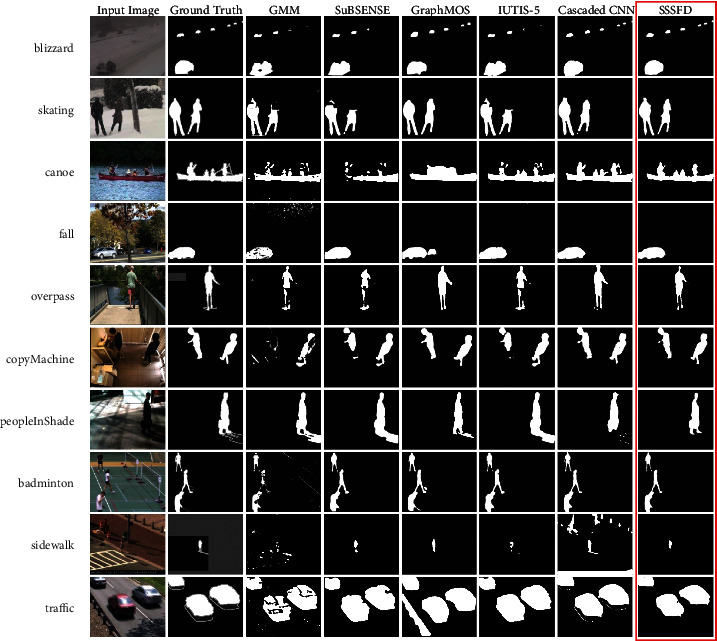
Qualitative results of the proposed method SSSFD and several state-of-the-art methods on CDNet2014. All the results are for the CDNet2014 dataset.

This section presents the exhaustive evaluation of SSSFD. The proposed algorithm is compared with eleven fundamental and state-of-the-art approaches, including unsupervised methods like GMM [44], KDE [11], ViBe [12], PAWCS [45], SuBSENSE [13], WeSamBE [14], SemanticBGS [8], and supervised methods such as IUTIS-5 [46], GraphMOS [47], BSUV-Net 2.0 [48], DVTN [49], Cascaded CNN [17], 3DCNN [35], and DeepBS [29]. Qualitative comparisons of the foreground results are shown in Figure 9, which involves ten challenging video sequences from the CDNet2014 dataset. Simultaneously, Table 4 reports the comparisons of the quantitative results of the SSSFD algorithm on category baseline, bad weather, dynamic background, shadow, and camera jitter. Note that the best and second-best performing algorithms are displayed in bold red and blue, respectively.

It is apparent from Figure 9 that SSSFD presents the best visual results, while GraphMOS and Cascaded CNN show competitive performance for these sequences. In the severe weather conditions of the “blizzard,” only SSSFD and Cascaded CNN detect six cars in far-end motion, and other algorithms lost foreground objects more or less. The sequence “fall” presents dynamic background variations challenge because of a large area of swaying leaves. There are many false positives in GMM's result because a single statistical model cannot describe the complex dynamic background. GraphMOS detects a stationary car in a parking lot as the foreground object, which may happen in some supervised algorithms. Benefiting from the supplementary information from the semantic background model, SSSFD effectively classifies stationary cars as the background. The “overpass” is another typical dynamic background sequence that contains a little shadow. Only SSSFD and GraphMOS can accurately locate pedestrians. However, due to the oversmoothing of GgraphMOS, the pedestrian is visually slightly rounder than the ground truth. In the “peopleInShade” and “badminton” sequences, a similar situation also occurs. The “sidewalk” is a video shot by a camera shakes violently. The camera jitter causes the position of all objects to move throughout the image, which is a massive challenge for any foreground detection algorithms. We observe that GMM cannot detect pedestrians because the variations of pixels in the picture do not follow a Gaussian distribution. The foreground masks provided by SuBSENSE and IUTIS-5 are also an incomplete part of the pedestrian's body. The detection results of SSSFD also lose part of the foreground, which is caused by the inability of the semantic segmentation network to segment small objects with irregular shapes accurately.

Table 4 proves that the performance of the deep learning-based foreground detection algorithm is much higher than that of the traditional unsupervised algorithm. Although it is controversial whether supervised foreground detection algorithms can be applied in actual video surveillance, we believe that high-performance supervised algorithms are helpful for some monitoring situations where foreground objects are relatively fixed. SSSFD achieves the best results in four out of five challenges, including baseline (0.9818), bad weather (0.9743), dynamic background (0.9756), and camera jitter (0.9819), while it gains encouraging performance for the remaining categories shadow (0.9840).

In conclusion, it is evident from the qualitative and quantitative results that SSSFD obtains better performance and is closer to the ground truth in most video sequences.

## 5. Conclusion

This paper proposes a foreground detection algorithm called SSSFD. It utilizes an unsupervised algorithm based on multiscale superpixel segmentation to acquire background subtraction results. We train a semantic segmentation network on a subset of manually selected frames from CDNet2014 and employ this network to perform semantic segmentation on input images. Updating the background model with fine foreground results composed of superpixel and semantic segmentation dramatically improves the accuracy of foreground detection. The experimental results on the CDNet2014 dataset verify the effectiveness of the proposed algorithm. SSSFD achieves state-of-the-art performance in visual and qualitative evaluation compared with other algorithms.

The limitation of the SSSFD algorithm is that the real-time performance is poor. This is because, first, multiscale superpixel segmentation takes up a lot of computational costs. Second, the processing speed of the high-precision semantic segmentation network is slow. Therefore, our future work will investigate more accurate and faster semantic segmentation networks and unsupervised background subtraction algorithms, as well as efficient fusion strategies for semantic information and background subtraction results.

## Figures and Tables

**Algorithm 1:**
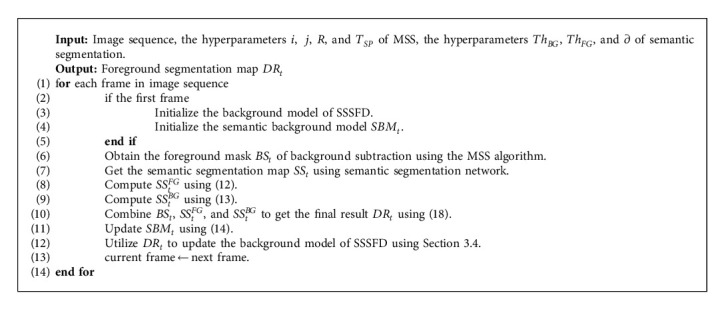
SSSFD algorithm

**Table 1:** The combination of three rules for SSSFD.

Input			Output
*SS*_*t*_^*BG*^(*x*) ≤ *Th*_*BG*_	*SS*_*t*_^*FG*^(*x*) ≥ *Th*_*FG*_	*BS*_*t*_(*x*)	*DR*_*t*_(*x*)
True	False	BG	BG
True	False	FG	BG
False	True	BG	FG
False	True	FG	FG
False	False	BG	BG
False	False	FG	FG

**Table 2:** The setting of hyperparameters.

*j*	*i*	*R*	*T*_*sp*_	*T*_*fg*_	*Th*_*BG*_	*Th*_*FG*_	*∂*
(8, 64, 256, 1024)	4	2	0.3	OTSU [43]	200	225	0.00024

**Table 3:** Comparison of detection results of SSN and SSSFD.

Category	Metrics								
	Recall			Precision			*F*-measure		
	SSN	SSSFD	Trend	SSN	SSSFD	Trend	SSN	SSSFD	Trend
Baseline	0.9668	0.9738	↑0.72%	0.9805	0.9900	↑0.97%	0.9736	0.9818	↑0.84%
Bad Weather	0.9649	0.9718	↑0.72%	0.9698	0.9768	↑0.72%	0.9673	0.9743	↑0.72%
Dynamic Background	0.9408	0.9695	↑3.05%	0.9645	0.9817	↑1.78%	0.9525	0.9756	↑2.42%
Shadow	0.9776	0.9839	↑0.64%	0.9838	0.9841	↑0.03%	0.9807	0.9840	↑0.34%
Camera Jitter	0.9715	0.9795	↑0.82%	0.9753	0.9843	↑0.92%	0.9734	0.9819	↑0.87%

**Table 4:** *F*-measure scores for each category produced by different foreground detection algorithms. The bold red entries indicate the best result in a given column, and the bold blue entries indicate the second-best result in a given column.

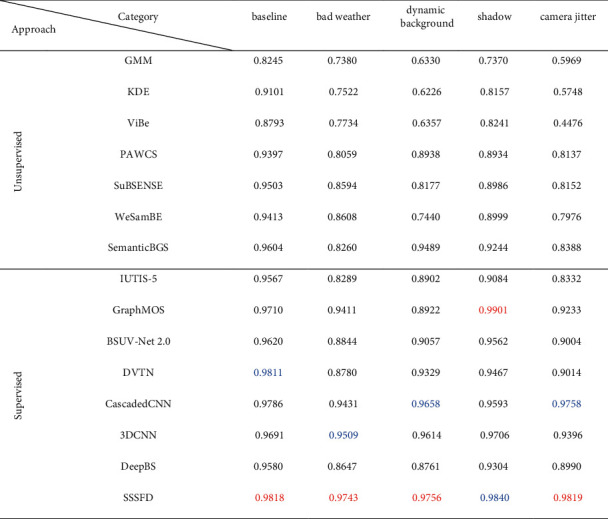

## Data Availability

The data used to support the findings of this study are available from the corresponding author upon request.
